# The problems with delay discounting: a critical review of current practices and clinical applications

**DOI:** 10.1017/S0033291721002282

**Published:** 2021-08

**Authors:** Allen J. Bailey, Ricardo J. Romeu, Peter R. Finn

**Affiliations:** Department of Psychological and Brain Sciences, Indiana University, Bloomington, IN, USA

**Keywords:** Alcohol, behavioral economics, construct validity, decision-making, delay discounting, impulsivity, RDoC, substance use, transdiagnostic

## Abstract

Delay discounting paradigms have gained widespread popularity across clinical research. Given the prevalence in the field, researchers have set lofty expectations for the importance of delay discounting as a key transdiagnostic process and a ‘core’ process underlying specific domains of dysfunction (e.g. addiction). We believe delay discounting has been prematurely reified as, in and of itself, a core process underlying psychological dysfunction, despite significant concerns with the construct validity of discounting rates. Specifically, high delay discounting rates are only modestly related to measures of psychological dysfunction and therefore are not ‘core’ to these more complex behavioral problems. Furthermore, discounting rates do not appear to be *specifically* related to any disorder(s) or dimension(s) of psychopathology. This raises fundamental concerns about the utility of discounting, if the measure is only loosely associated with most forms of psychopathology. This stands in striking contrast to claims that discounting can serve as a ‘marker’ for specific disorders, despite never demonstrating adequate sensitivity or specificity for any disorder that we are aware of. Finally, empirical evidence does not support the generalizability of discounting rates to other decisions made either in the lab or in the real-world, and therefore discounting rates cannot and should not serve as a summary measure of an individual's decision-making patterns. We provide recommendations for improving future delay discounting research, but also strongly encourage researchers to consider whether the empirical evidence supports the field's hyper-focus on discounting.

## Introduction

Delay discounting is a staple for examining intertemporal choice (ITC) in clinical research. In fact, a Google Scholar search for ‘delay discounting’ gives hundreds of results in the past 5 years alone. Delay discounting rates (of rewards) intend to measure the extent to which a future reward (or incentive) is reduced in value relative to an immediate reward as a function of the temporal delay of the future reward. Delay discounting paradigms have enjoyed widespread popularity in the field. For example, there are meta-analyses examining the association between performance on delay discounting tasks in healthy controls compared to those with a range of clinical disorders, such as addictive disorders (MacKillop et al., 2011), attention deficit hyperactivity disorder (Jackson & MacKillop, 2016), and other disorders including depression, disordered eating, and psychotic disorders (Amlung et al., 2019). Steeper delay discounting rates have been associated with so many different disorders that it has increasingly been discussed as a possible transdiagnostic process underlying a variety of common mental health problems (Amlung et al., 2019; Bickel et al., 2019; Finn, Gunn, & Gerst, 2015; Lempert, Steinglass, Pinto, Kable, & Simpson, 2019). Although delay discounting is discussed as a potential key transdiagnostic process in psychopathology, we believe, given the available research, it remains difficult to even describe what process these rates capture and how central it might be in psychopathology.

The premise of this paper is that the large body of current, as well as future, research on the relationships between decision-making, ITC, and psychopathology will have more value if there is a greater understanding of the significant problems and limitations in delay discounting research up to this point. We posit that there has been a premature theoretical acceptance of delay discounting as, in and of itself, a core process underlying psychological dysfunction. Furthermore, we believe there is a growing disconnect between the empirical evidence of the utility of delay discounting in clinical science and both the incredible popularity of the task and the lofty goals for its usage in clinical research.

For example, researchers continue to promote the importance and centrality of delay discounting in clinical disorders, including labeling discounting as a core trans-disease (Bickel & Mueller, 2009; Bickel, Jarmolowicz, Mueller, Koffarnus, & Gatchalian, 2012) and/or transdiagnostic (Amlung et al., 2019) process, or that delay discounting would fulfill the promises of the Research Domain Criteria (RDoC; Insel et al., 2010) initiative (Lempert et al., 2019). We certainly applaud research that aims to study processes across multiple disorders and embraces the dimensional approaches championed by the RDoC. However, our primary concern is that delay discounting, and subsequently the discounting rates obtained from the tasks, have been conflated with the actual underlying *latent* construct of interest (i.e. impulsive choice). Delay discounting is at best a candidate paradigm at one level of analysis to examine some, but certainly not all, processes that influence ITC patterns (a view shared by Dai & Busemeyer, 2014; Read, Frederick, & Scholten, 2013). In stark contrast, ITC, in our view, is a broad label to describe the complex and multifaceted processes that contribute to how individuals make decisions in the real-world related to maximizing benefits over time. Although we certainly concede it is unreasonable to expect any measure to capture ITC processes entirely, we do believe it is vital to stringently examine whether a popular measure provides enough information generalizable to the actual construct of interest. This is not merely a semantic argument; this premature reification has led to a drastic hyper-focus on a particular task that has yet, despite its popularity, to show substantial utility in clinical science. This paper will describe three key issues:

1. Discounting research has not provided adequate evidence of convergent validity to provide confidence in how to characterize discounting rates using other validated constructs.
2. Discounting rates also have not shown evidence of divergent validity when examining the association between discounting rates and other well-validated psychological measures, which presents another fundamental theoretical concern for how to interpret these rates.
3. The generalizability of delay discounting rates to other types of decisions, laboratory or real-world, is *extremely* limited. Therefore, discounting rates should not be considered a generalizable summary of an individual's decision-making or ITC patterns.

### Convergent validity concerns

Despite hundreds of studies, discounting rates are poorly understood in terms of basic convergent validity with well-validated psychological measures. For example, delay discounting tasks have enjoyed widespread use in the study of addictive behaviors with meta-analyses finding that groups with addictive behaviors tend to discount at higher rates than healthy controls (MacKillop et al., 2011) and that discounting rates are related to continuous measures of addiction severity (Amlung, Vedelago, Acker, Balodis, & MacKillop, 2017). Differences in delay discounting rates are hypothesized to reflect variations in self-control, where higher discounting rates are thought to reflect deficits in self-control (or impulsivity) that lead individuals to choose smaller immediate options (e.g. intoxication) over long-term larger rewards (e.g. gainful employment). Although this explanation certainly has face value in its relationship to substance use pathology, the empirical findings have struggled to support this interpretation. Discounting rates are only modestly related to addiction severity based on meta-analysis (*r* = 0.14; Amlung et al., 2017), which must call into question how ‘core’ this process can be if it accounts for ~2% of the variance of symptom severity. Moreover, discounting rates are largely uncorrelated with other measures of impulsivity, which call into question the hypothesized relationship between discounting and addiction (Amlung et al., 2017; Kvam, Romeu, Turner, Vassileva, & Busemeyer, 2021; MacKillop et al., 2016; Sharma, Markon, & Clark, 2014). Delay discounting rates are not synonymous with impulsive decision-making as they are sometimes used in the literature. In fact, it does not appear that the constructs are even closely related. Rather, impulsivity and poor self-control in the context of decision-making reflect numerous processes, which clearly are not captured by delay discounting tasks.

Furthermore, discounting rates, to our knowledge, have not shown strong and replicable associations with any relevant psychological phenomena to provide a compelling explanation of what these rates characterize. For example, a recent large sample study found that discounting rates were uncorrelated or only modestly correlated (*r* values <0.20) with all tested cognitive abilities and personality measures, and that these correlations became even lower when controlling for income and education (Yeh, Myerson, & Green, 2020). This is consistent with previous literature that has shown only modest associations between discounting rates and measures of executive function (Bobova, Finn, Rickert, & Lucas, 2009; Weatherly & Ferraro, 2011) and personality (Bobova et al., 2009; Hirsh, Morisano, & Peterson, 2008). We believe Yeh et al. (2020) provided a very well thought-out and insightful study, however we take issue with some of the broader conclusions given the presented results, specifically:

The current findings suggest that steep discounting, a behavior strongly related to behavioral problems, is not simply an indicator of generally poor cognitive functioning or a measure of impulsiveness in healthy young adults as assessed by personality tests, but is an important individual difference characteristic in its own right. (p. 8)

As previously stated, we do not believe the evidence supports the strength of a relationship between discounting and behavior problems; we believe it is more accurate to say there is simply a modest reliable association. Furthermore, and most importantly, we are unsure what makes discounting an ‘important’ individual difference until the measure demonstrates its importance above and beyond existing measures (incremental validity). We agree it is positive that discounting is not simply a redundant measure of a construct with already well-established measures (e.g. general intelligence). However, we believe discounting is so poorly characterized that it is essentially impossible to even describe what discounting rates mean in terms of well-established constructs, given its poor relationship to other impulsive decision tasks, impulsive personality, and executive functioning measures. We do not want to overstate our case and claim that the signal being detected through discounting tasks as useless; however, we believe researchers must be aware about how little we know about what performance on this task means theoretically. Moreover, the burden of proof must be on the researchers who claim the centrality and usefulness of discounting to provide concrete and empirical examples of its utility.

In the same vein, although delay discounting rates have been shown to be significantly influenced by experimental manipulations (Read et al., 2013; Wilson & Daly, 2004), the processes responsible for these changes are unknown. Rung and Madden (2018) provided a review and meta-analysis of 92 published studies that examined methods to reduce discounting rates and reported that although many techniques succeed in reducing discounting rates (with substantial variability), there is no clear picture of *how* these manipulations influence discounting rates, or whether these changes coincide with reductions in impulsive decision-making more broadly. Importantly, research has demonstrated that discounting rates can be effectively influenced by a plethora of superficial task characteristics (Read et al., 2013) and therefore any observed changes in discounting rates must be closely examined. Therefore, although task manipulations can be valuable to probe a task to gain a better understanding of the underlying processes, after nearly 100 studies about reducing discounting rates, we still have not gained much general knowledge about how to characterize the signal being picked up through the task. Taken together, discounting rates stand on shaky theoretical ground, and subsequently, studies that attempt to manipulate discounting rates have struggled to illuminate the processes captured by the task.

In summary, we believe, given the enormous volume of discounting data, we know discouragingly little about the processes underlying the task or even how to characterize the rates in terms of validated constructs. If modest correlations are somewhat expected between laboratory tasks and complex behaviors (e.g. real-world substance use), then our theories must match this theoretical complexity. Discounting cannot be both too ‘basic’ an assessment to be associated strongly with the measures of complex behaviors (i.e. substance use), but also be a ‘core’ process underlying multiple disorders. Furthermore, we cannot let the simplicity and face validity of the task distract us from rigorously testing the task. For example, perhaps discounting taps into a certain basic cognitive process that serves as an underlying risk factor for impulsive ITCs and then consequently substance use risk. Then research should aim to find a measure, or more likely measures, that illuminate impulsive ITC patterns more broadly. This would return focus to the actual construct of interest (i.e. generalizable processes in impulsive choice) that serve as more direct risk factors for psychopathology. In this vein, we agree with recommendations in Sharma et al. (2014) that researchers should aim to connect their laboratory studies as much as possible to real-world decisions and behaviors. We cannot simply infer a face-valid cascade from a very basic assessment to complex behaviors.

### Divergent validity concerns

Despite its face-valid, hypothesized connection with problematic substance use, research has demonstrated that steeper discounting of rewards compared to controls is associated with depression, bipolar, schizophrenia, borderline personality disorder, bulimia nervosa, binge-eating disorder (Amlung et al., 2019), and lower intelligence (Bailey, Gerst, & Finn, 2020; Shamosh & Gray, 2008). Notably, effect sizes are comparable when contrasting controls to clinical populations, although effects appear slightly larger in more severe clinical populations such as those with psychotic-spectrum disorders or illicit substance use disorders (Amlung et al., 2019; MacKillop et al., 2011). This lack of divergent validity is cause for significant concern for interpreting these abundant group differences. Although self-control deficits are a common interpretation for the relationship between steeper delay discounting rates and externalizing behavior, this interpretation seems unlikely to apply to all, or even most, disorders associated with high discounting rates (e.g. depression). To be clear, it is plausible that disparate pathological processes could result in steeper discounting rates in different disorders (i.e. ‘equifinality’; Cicchetti & Rogosch, 1996). However, this is an empirical question that requires more research into the different processes, factors, and mechanisms that contribute to variations in delay discounting rates across clinical samples (Story, Moutoussis, & Dolan, 2016). Until theories about specific mechanisms are formally tested, researchers should be wary of untested, usually ad-hoc explanations of the observed group differences.

Perhaps steeper discounting rates are simply associated with the general psychopathology factor (Caspi et al., 2014) and an underlying risk factor for most psychological disorders. As reviewed above, discounting rates appear to have a mostly nonspecific relationship to overall psychological severity. This drastically changes the interpretations provided in the literature, which tend to have diagnosis- or dimension-specific explanations with almost no empirical backing. This lack of divergent validity leads to possibly sobering questions about the utility of delay discounting rates. For example, if assessed in a group of individuals with unclear diagnostic status, delay discounting rates would be essentially useless in predicting diagnostic status [e.g. alcohol use disorder (AUD) *v.* depression]. This stands in striking contrast to claims that discounting can and does serve as a ‘biomarker’ (Kwako, Bickel, & Goldman, 2018) or ‘behavioral marker’ (Athamneh et al., 2020; Bickel et al., 2012; Bickel, Koffarnus, Moody, & Wilson, 2014; Turner, Athamneh, Basso, & Bickel, 2021) given it wholly fails to be either adequately sensitive or specific to *any* psychological phenomena to warrant such status. However, in a highly cited review, Bickel et al. (2014) come to drastically different conclusions saying ‘Our review suggests that temporal discounting (1) identifies individuals who are drug-dependent, (2) identifies those at risk of developing drug dependence, (3) acts as a gauge of addiction severity, (4) correlates with all stages of addiction development…’ (abstract). We agree discounting is modestly associated with many aspects of addiction; however, this in no way indicates that discounting can reliably identify any clinical population. Furthermore, commonly cited studies that make such strong claims of the utility of discounting rates to predict future substance use only reported modest to very modest associations (Audrain-McGovern et al., 2009; Fernie et al., 2013; Khurana et al., 2013). Importantly, for discounting rates to be valuable in terms of identification of clinical populations, it would need to show incremental validity over already existing measures. Framed this way, it should be obvious that one would never select to screen participants or patients for AUD using a discounting task instead of, for example, the Alcohol Use Disorder Identification Test (AUDIT), a brief, freely available, self-report measure, which across studies has shown a median sensitivity of 0.86 and specificity of 0.89 for identifying AUD (Reinert & Allen, 2002). We certainly understand the tremendous value of laboratory tasks to provide information that self-report measures cannot, however it is important to be realistic about the utility of each in different situations. In summary, modest associations with criteria of interest (e.g. addiction severity) do not qualify as strong evidence for the importance of that measure. Discounting rates must demonstrate that they are highly predictive of criteria of interest or that they outperform existing measures to have substantial predictive value. Moving forward, we believe the field must be much more stringent examining claims of the usefulness of discounting rates in the face of mounting evidence to the contrary.

We believe the delay discounting literature has failed to adequately examine delay discounting rates from a classic construct validity standpoint (Cronbach & Meehl, 1955). Despite face-valid explanations for discounting rates and the observed group differences in clinical populations, the empirical data are simply not there to provide confidence in these explanations. We again concur with Sharma et al. (2014) in stressing that researchers apply the same psychometric and construct validity considerations to behavioral tasks as they do self-report measures. Face-valid behavioral tasks should not be exempt from empirically demonstrating construct validity.

### The generalizability (or lack thereof) of discounting rates

Evidence for claims of delay discounting serving as a generalizable measure of ITC is scant. Although researchers have demonstrated discounting rates of rewards are relatively stable over time (Odum, 2011; Ohmura, Takahashi, Kitamura, & Wehr, 2006), the empirical evidence does not suggest that discounting rates are highly informative about other decisions. Research has shown that a discounting rate from a certain discounting task is not highly informative even of performance on other discounting tasks. Weatherly and colleagues (Weatherly & Terrell, 2010; Weatherly, Terrell, & Derenne, 2010) performed exploratory and confirmatory factor analyses to show that discounting rates across five commodities are *not* best explained by a single discounting factor, a result congruent with the modeling results in Kvam et al. (2021). Furthermore, there is evidence that discounting rates can be heavily influenced by experimental manipulations (Rung & Madden, 2018) and task framing (Read et al., 2013). Therefore, it is possible that individuals have a trait-like baseline discounting rate that can be influenced by manipulations/circumstances (Peters, Miedl, & Büchel, 2012); however, it is unclear how one would identify this baseline or whether this baseline value has significant predictive value. Most importantly, this means that even within the rather limited scope of delay discounting of rewards, a single discounting rate provides only modest information about performance on *very* similar tasks. Therefore, delay discounting cannot serve as a summary measure for general ITC or decision-making patterns, which includes discounting across and between different commodities (Story et al., 2016), contexts, probabilistic assessment, and discounting of losses (Bailey, Gerst, & Finn, 2018), among other processes. We believe the generalizability and value of discounting rates has been drastically overstated given their inability to robustly predict other decisions made either in the lab or real life.

Finally, despite limited evidence on the generalizability of discounting rates, some researchers have called for interventions to reduce steepness of delay discounting rates as a prevention or intervention for those at risk for addiction (e.g. Bickel et al., 2017; Gray & MacKillop, 2015; Mahalingam, Stillwell, Kosinski, Rust, & Kogan, 2014; Volkow & Baler, 2015), whereas other researchers have used decreased discounting rates as the primary outcome measure in an intervention study (e.g. working memory training; Bickel, Yi, Landes, Hill, & Baxter, 2011). In clinical disorders, the problem is that impulsive choices increase the likelihood of maladaptive behavior (like problematic substance use), or behavior that does not optimize outcomes (e.g. low achievement), not that they have higher rates on a delay discounting task. We hope we have made the case that these admirable endeavors are overly focused on the singular task at the expense of the more important construct(s). Designing interventions to address performance on a single task is similar to instructors teaching the skills of a standardized test at the expense of the knowledge base the test was meant to assess. In this case, the assessment (discounting rates) is not even robustly related to the criteria of interest (real-world behavior or symptomology) and therefore, in our estimation, does not appear to be a logical target of intervention.

## Future directions and recommendations

Given the fundamental issues with delay discounting, we believe it is clear the necessity to improve and innovate our research programs related to ITC processes in clinical populations. We currently have hundreds of delay discounting studies in clinical science and seemingly little generalizable knowledge beyond a disorganized collection of unexplained group differences. We will conclude with a brief description of some suggestions for improvement in the field. We will discuss:

1. Improving the measurement of discounting rates;
2. Suggestions to improve our understanding of discounting rates through mechanism-focused research; and
3. Innovating new paradigms to assess processes related to ITC beyond discounting rates.

Importantly, these suggestions will encompass only a small set of possible improvements.

### Improving measurement of discounting

Although our primary concerns with delay discounting practices are theoretical, improving the measurement of delay discounting rates may provide a fruitful avenue to improve our understanding of the task. Although a specific review and explanation of measurement concerns is beyond the scope of the current paper, we have several broad recommendations. First, given the significant concerns raised in the current study, we have strong reservations about attempts to shorten existing discounting measures (e.g. Koffarnus & Bickel, 2014). Given that discounting rates are poorly understood in essentially all aspects of construct validity and show modest associations at best with external criteria, we do not understand why researchers would embrace a less reliable version of the task. For example, Koffarnus and Bickel (2014) reported a correlation of 0.67 between their five-trial adjusting discounting task (i.e. short-form measure) and a longer adjusting amount discounting task (i.e. original long-form). This means the short-form of the measure only predicts 45% of the variance of the original form, which based on meta-analysis is only expected to correlate with most criteria of interest around *r* < 0.20. We believe this could decrease reproducibility and increase spurious findings that will not help the field wrestle with the challenges reviewed in the current paper. These concepts are discussed in detail by Smith, McCarthy, and Anderson (2000) in relation to self-report measures, specifically the dangers of developing a short-form of a measure that itself is insufficiently validated.

In fact, we would suggest an opposite course of action. We believe researchers should look to embrace assessment and scoring methods that collect sufficient amounts of data and then model *all* the collected trial-level data (Dai & Busemeyer, 2014; Dai, Gunn, Gerst, Busemeyer, & Finn, 2016; Kvam et al., 2021; Molloy et al., 2020). This is in contrast to the majority of discounting scoring practices that rely on indifference points for each time-delay (e.g. 1 week, 1 month). Indeed, Kvam et al. (2021) provide code that researchers can use or adapt for their own purposes that implements the ‘direct difference’ model (Dai & Busemeyer, 2014). Interestingly, the ‘direct difference' model not only models all collected trial-level decisions, but there are also versions that can incorporate decision reaction time to further elucidate decision-making processes such as difficulty of deliberation (Dai & Busemeyer, 2014). If the researcher still wishes to use the standard hyperbolic model, there are estimation procedures that are not solely reliant on indifference points and model all collected data (Molloy et al., 2020; Vincent, 2016). Molloy et al. (2020) also provide usable codes for interested researchers.

We also warn that overly focusing on modeling the data, without regard to the theoretical concerns attached to those data, comes with significant drawbacks, and can even compound the issues discussed thus far. For instance, Johnson and Bickel (2008) recommend that researchers exclude data which do not have sufficiently decreasing indifference points. These criteria were suggested to improve fitting procedures when using the hyperbolic model. However, the danger is that these criteria lead to researchers throwing out data which do not conform to the hyperbolic model, and thus the model is tested only on data that are chosen to conform to it. Smith, Lawyer, and Swift (2018) found that close to a *fifth* of discounting data is discarded per study using the Johnson and Bickel (2008) criteria. Although these criteria are touted as suggestions, their widespread use in the literature suggests they are closer to conventions. Most importantly, they create a vicious circle in which the hyperbolic model has been lauded as the proper model for discounting, using only evidence that happens to favor the hyperbolic model. In other words, the excuse of having a ‘viable’ model is used to justify unscientific practices in modeling discounting data, specifically attempting to change the phenomena to fit the preferred model. This is especially problematic when studying clinical populations, whose decisions may not seem immediately ‘rational’ and where response ‘abnormality’ is the rule, not the exception. Furthermore, studies have shown that the hyperbolic model is not the most appropriate model for all participants and therefore it is not appropriate to assume all participants' performance must conform to an *a priori* model (Franck, Koffarnus, House, & Bickel, 2015; Gilroy, Franck, & Hantula, 2017). Similarly, Cheng and González-Vallejo (2016) demonstrated that the hyperbolic model may have significant performance concerns when discounting tasks are not presented in the traditional ‘titration’ procedure. Finally, acquiring the ‘correct’ or ‘best’ model for a given task is a goal that is secondary to making sure the task is actually a valid one for the construct at hand; indeed, a model's usefulness is always bounded above by the data's validity. We thus urge researchers to reconsider using the Johnson and Bickel (2008) criteria in the future and instead to return their focus to providing models that elucidate meaningful and generalizable psychological processes.

In summary, given the theoretical concerns described in the current paper, we believe researchers should be actively concerned with improving the quality of their measurement and not embracing practices that could increase measurement concerns.

### Improving delay discounting construct validity

Researchers have not provided adequate evidence to properly characterize discounting rates to justify the majority of theoretical explanations. This should lead to an increase in scrutiny over studies providing group differences that are not further explained by empirical analyses. For example, a group difference in discounting between those with and without AUD should not conclude with an ad-hoc explanation of self-control deficits; this should be empirically corroborated with established measures of self-control (see Sharma et al., 2014). Furthermore, discounting must show robust, not just statistically significant, associations to claim strong relationships with constructs of interest. Relatedly, the use of extended task batteries and multivariate approaches would certainly yield a better understanding of how delay discounting performance relates to other established constructs. Snyder, Miyake, and Hankin (2015) provide a useful roadmap for ways of improving construct validity in the assessment of executive functioning, and many of the suggestions are germane to the current discussion. Just like executive functioning, ITC will never be captured by a single task. However, multivariate approaches can assist in elucidating the structure underlying many tasks and related psychological measures. Furthermore, these multivariate approaches can mitigate concerns from the ‘task impurity’ problem (Miyake & Friedman, 2012; Snyder et al., 2015), or the concern that any individual task score contains systematic variance *not* related to the construct of interest, but related to the task. Multivariate approaches, such as MacKillop et al. (2016) and Weatherly and Terrell (2010), especially when combined with cognitive modeling approaches (Dai & Busemeyer, 2014; Kvam et al., 2021; Molloy et al., 2020), can help researchers elucidate common processes underlying delay discounting and other relevant psychosocial measures.

Relatedly, studies with longitudinal data, especially intervention studies, must provide more rigorous support for interpretations related to delay discounting. For example, Bickel et al. (2011) showed that discounting rates were lowered significantly in stimulant abusers who received a working memory training protocol compared to those who received a control condition. Beyond methodological concerns with this and other working memory studies (see Gunn, Gerst, Wiemers, Redick, & Finn, 2018), a major concern is that many studies provide limited corroborating analyses to contextualize these findings. Specifically, if steeper delay discounting is related to executive functioning and consequently self-control, then it stands to reason that working memory training could improve task performance. However, providing group differences across conditions (i.e. working memory training *v.* control) does not provide strong evidence of the proposed mechanism. If the above hypothesis about the benefits of working memory training is true, then individuals who benefit the most from working memory training should be the same individuals who show the most improvement in delay discounting tasks. That is, researchers should focus on specifying the degree of change, if any, rather than a simple ‘present–absent’ assessment. Moreover, the same criticism applies to linking changes in delay discounting rates to changes in behavior such as drinking patterns. For example, studies that observe changes in discounting and changes in other types of decisions/behaviors after an intervention (Athamneh, Stein, & Bickel, 2019; Mellis et al., 2018; Snider, LaConte, & Bickel, 2016; Stein et al., 2017) are not adequate evidence to conclude any causal relationship, as Stein et al. (2017) suggested: ‘Accumulating laboratory-based evidence indicates that reducing delay discounting (devaluation of delayed outcomes) with the use of episodic future thinking (EFT; mental simulation of future events) improves dietary decision-making and other maladaptive behaviors’ (abstract). We must be more stringent about implying or reporting causal mechanisms that have not been empirically established, as mounting evidence indicates we do not have a strong grasp of the processes underlying discounting tasks or how these processes relate to other decisions.

Finally, there remains a muddled picture of how delay discounting relates to dysfunction, despite the abundance of studies. As described above, the lack of diagnostic/dimension specificity of delay discounting findings is of fundamental concern. Observing a relationship across many disorders is not convincing evidence of an important trans-disease process when those relationships are uniformly weak from a statistical perspective and poorly understood from a theory-development perspective. Despite efforts to characterize discounting rates as a transdiagnostic process, there remains minimal evidence to what the process is and exactly how it unifies the breadth of dysfunctions associated to it. Hierarchical multivariate approaches to modeling and conceptualizing psychological dysfunction, such as the Hierarchical Taxonomy of Psychopathology (HiTOP; Kotov et al., 2017), have shown tremendous benefits in empirically examining transdiagnostic processes. Previous research, for example, has shown that steeper delay discounting rates were associated with the general externalizing dimension of psychopathology, and *not* differentially related to specific externalizing disorders (Finn et al., 2015). This research must be continued and broadened substantially; indeed, current findings suggest steep delay discounting relates to processes that contribute to a large array of dysfunction even broader than the externalizing dimension. Practically speaking, delay discounting studies analyzing only a minimally diverse diagnostic sample (e.g. diagnostic group ‘X’ *v.* controls) will continue to have extreme difficulties in illuminating how to understand this task in clinical science at large.

### Moving beyond discounting and embracing ITC

We described some methods to improve research related to delay discounting and clinical populations. However, our primary recommendation would be to heavily consider embracing alternative and creative assessments of ITC beyond delay discounting tasks. Traditional delay discounting tasks have significant limitations even when following all recommendations in the current paper. As discussed in Sharma et al. (2014), researchers should aim to connect their laboratory tasks to real-world decisions/behaviors as much as possible. For example, Finn, Gerst, Lake, and Bogg (2017) asked a high externalizing sample of students to make decisions related to attending/drinking at certain events that varied in terms of incentives (e.g. friends at the party), and disincentives (e.g. you have a test the next day) and found that individuals with antisocial personality traits where more likely to be uninfluenced by disincentive levels when making decisions. This paradigm has many similarities to traditional delay discounting tasks, but provides added complexity to examine specific processes related to externalizing psychopathology and gives the decisions made in the task more external validity. It is possible that more complex and ecologically valid tasks are needed to ‘bridge’ the gap between very basic tasks such as traditional monetary discounting and complex behaviors such as substance use. Moreover, we urge the field to focus on providing tasks and models that we can empirically demonstrate are *strongly* related to clinical phenomena. We cannot be overly enamored with one face-valid task we believe will solve all these problems for us. We strongly encourage researchers to more carefully examine how well narratives around the utility of discounting rates are backed by strong empirical support. Despite centering on delay discounting in the current paper, we believe these principles apply to the use of decision-making paradigms in clinical science at large.
